# Carbohydrate structures of human alpha-fetoprotein of patients with hepatocellular carcinoma: presence of fucosylated and non-fucosylated triantennary glycans.

**DOI:** 10.1038/bjc.1993.91

**Published:** 1993-03

**Authors:** Y. Aoyagi, Y. Suzuki, K. Igarashi, A. Saitoh, M. Oguro, T. Yokota, S. Mori, T. Suda, M. Isemura, H. Asakura

**Affiliations:** Department of Internal Medicine, Niigata University School of Medicine, Japan.

## Abstract

**Images:**


Br. J. Cancer (1993), 67, 486-492 © Macmillan Press Ltd., 1993

### Carbohydrate structures of human α-fetoprotein of patients with hepatocellular carcinoma: presence of fucosylated and non-fucosylated triantennary glycans

Y. Aoyagi[1], Y. Suzuki[1], K. Igarashi[1], A. Saitoh[1], M. Oguro[1], T. Yokota[1], S. Mori[1], T. Suda[1], M. Isemura[2] & H. Asakura[1]

[1]*The Third Division, Department of Internal Medicine, Niigata University School of Medicine, Asahimachi Dori-1-Bancho, 757, Niigata 951 and* [2]*Laboratory of Biochemistry, School of Food and Nutritional Sciences, The University of Shizuoka, Shizuoka 422, Japan.*

**Summary** Chemical structures of the sugar chains of various human α-fetoprotein (AFP) species with different affinity for Concanavalin A (Con A) and *Lens culinaris* agglutinin (LCA) were examined by pyridylamination of their oligosaccharides and stepwise exoglycosidase digestion. Using reversed-phase and size-fractionation high performance liquid chromatography systems we identified six pyridylamino-sugar chains. The Con A-reactive and LCA-nonreactive species of AFP from patients with hepatocellular carcinoma contained a biantennary sugar chain, and the Con A-reactive and LCA-reactive species had a biantennary one with a fucose residue at the innermost N-acetylglucosamine residue. The Con A-nonreactive and LCA-reactive species contained a biantennary sugar chain both with a bisecting-N-acetylglucosamine residue at the trimannosyl core and with a focuse residue at the innermost N-acetylglucosamine residue. The Con A-nonreactive and LCA-nonreactive species contained a fucosylated triantennary sugar chain as a major component, and two minor components: a triantennary sugar chain and a biantennary sugar chain with a bisecting-N-acetylglucosamine residue at the trimannosyl core. Thus, the fucosylated and non-fucosylated triantennary sugar chains were newly identified in human AFP. Essentially identical results were obtained for AFP from the patient with gallbladder carcinoma which metastasises to the liver. These results indicate that the increment in fucosylation and branching to form new antennae is a characteristic feature of the carbohydrate chains of AFP from patients with neoplastic diseases of the liver.

The measurement of the serum concentration of α-fetoprotein (AFP) has been used extensively for the early diagnosis of hepatocellular carcinoma (HCC) during a follow-up process of chronic liver diseases (Abelev, 1986; O'Conor *et al.*, 1970; Nishi & Hirai, 1973). However, the serum concentration of AFP also increases in patients with carcinomas of digestive organs which metastasise to the liver (Alpert *et al.*, 1971; Mcintire *et al.*, 1975) and with nonneoplastic diseases of the liver (Karvountzis & Redeker, 1974; Alpert & Feller, 1978).

Several investigators and we have reported that the reactivity of AFP with *Lens culinaris* agglutinin (LCA) and concanavalin A (Con A) is a good measure for distinction between HCC, nonneoplastic diseases of the liver and carcinomas of digestive organs which metastasise to the liver (Ruoslahti *et al.*, 1978; Breborowicz *et al.*, 1981; Ishiguro *et al.*, 1985; Taketa & Hirai, 1989; Aoyagi *et al.*, 1984, 1991). The molecular basis for the different affinity of AFP for LCA and Con A is presumed to be the difference in fucosylation at the innermost N-acetylglucosamine residue and bisecting-glucosaminylation in the biantennary carbohydrate chain (Aoyagi *et al.*, 1985). However, precise chemical structures of sugar chains in these molecular species of AFP have not been fully determined.

In this manuscript, we report the chemical structures of sugar chains of human AFP molecular species with special reference to reactivity to lectins and the disease category.

Correspondence: Y. Aoyagi.

In this paper, the terms of sugar chains are used for those with structures as follows. Biantennary, Galβ1-4GlcNAcβ1-2 Manα1-6 (Galβ1-4Glc NAcβ1-2 Manα1-3) Manβ1-4 GlcNAcβ1-4 GlcNAc-PA; agalactobiantennary, GlcNAcβ1-2 Manα1-6) GlcNAcβ1-2 Manα 1-3) Manβ1-4 GlcNAcβ1-4 GlcNAc-PA; trimannosyl, Manα1-6 (Manα1-3) Manβ1-4 GlcNAcβ1-4 GlcNAc-PA; fucosylated biantennary, Galβ1-4 GlcNAcβ1-2 Manα1-6 (Galβ1-4 GlcNAcβ1-2 Manα1-3) Manβ1-4 GlcNAcβ1-4 (Fucα1-6) GlcNAc-PA; fucosylated and N-acetylglucosaminylated biantennary, Galβ1-4 GlcNAcβ1-2 Manα1-6 (GlcNAcβ1-4) (Galβ1-4 GlcNAcβ1-2 Manα1-3) Manβ1-4 Glc NAcβ1-4 (Fucα1-6) GlcNAc-PA; N-acetylglucosaminylated biantennary, Galβ1-4 GlcNAcβ1-2 Manα1-6 (GlcNAcβ1-4) (Galβ1-4 GlcNAcβ1-2 Manα1-3) Manβ1-4 GlcNAcβ1-4 GlcNAc-PA; fucosylated triantennary, Galβ1-4 GlcNAcβ1-4 (Galβ1-4 GlcNAcβ1-2) Manα1-3 (Galβ1-4 GlcNAcβ1-2 Manα1-6) Manβ1-4 GlcNAcβ1-4 (Fucα1-6) GlcNAc-PA; triantennary, Galβ1-4 GlcNAcβ1-4 (Galβ1-4 GlcNAcβ1-2) Manα1-3 (Galβ1-4 GlcNAcβ1-2 Manα1-6) Manβ1-4 GlcNAcβ1-4 GlcNAc-PA; agalactotriantennary, GlcNAcβ1-4 (Glc NAcβ1-2) Manα1-3 (GlcNAcβ1-2 Manα1-6) Manβ1-4 GlcNAcβ1-4 GlcNAc-PA.

Received 5 June 1992; and in revised form 8 October 1992.

### Materials and methods

### Materials

AFP specimens were prepared from serum samples of two patients with HCC and a patient with gallbladder carcinoma which metastasises to the liver by affinity chromatography as described previously (Aoyagi *et al.*, 1977). Con A- and LCA-Sepharose 4B beads were obtained from Pharmacia Fine Chemicals, Uppsala, Sweden. Lyophilised Con A and LCA, bovine epididymal α-L-fucosidase, Jack beans β-N-acetylglucosaminidase, and neuraminidase from *Clostridium perfringens*, type X, were from Sigma Chemical Co., St. Louis, MO, USA. β-Galactosidase from *E. coli* was purchased from Boehringer Mannheim, Germany. Authentic pyridylamino (PA)-oligosaccharide standards were from Takara Shuzo Co., Ltd. Kyoto, Japan, and their structures were verified by $^{1}$H-nuclear magnetic resonance by the manufacturer. Anhydrous hydrazine was from Pierce Chemical Company, Rockford, Ill, USA and 2-aminopyridine was from Nacalai Tesque, Kyoto, Japan. Sodium cyanoborohydride was from Aldrich Chem. Co., Milw., WI, USA. The other reagents were of analytical grade.

### Methods

*Sodium dodecyl sulfate-polyacrylamide gel electrophoresis*

The purity of the AFP prepared by affinity chromatography was checked by sodium dodecyl sulfate-polyacrylamide gel electrophoresis according to the method of Laemmli (1970).

*Isolation of AFP species with different affinity for Con A and LCA*

Four AFP species, i.e., Con A-nonreactive and LCA-nonreactive {Con A(−)/LCA(−)}, Con A-nonreactive and LCA-reactive {Con A(−)/LCA(+)}, Con A-reactive and LCA-nonreactive {Con A(+)/LCA(−)}, Con A-reactive and LCA-reactive {Con A(+)/LCA(+)} species were obtained from each AFP sample with Con A- and LCA-Sepharose 4B as described previously (Aoyagi *et al.*, 1985).

*Crossed immunoaffinoelectrophoresis*

Purity of each molecular species of AFP with different affinity for Con A and LCA was examined by crossed immunoaffinoelectrophoresis as described previously (Aoyagi *et al.*, 1984).

*Preparation of PA-sugar chains*

Sugar chains of AFP samples were released by hydrazinolysis at 100°C for 10 h and free amino groups were N-acetylated. Then, the free oligosaccharides were reductively aminated with a fluorescent reagent, 2-aminopyridine, by use of sodium cyanoborohydride, and PA-derivatives of each oligosaccharide preparation were fractionated by Sephadex G-15 gel filtration (1 × 50 cm) according to the method described previously (Hase *et al.*, 1984; Yamamoto *et al.*, 1989).

*Glycosidase digestion of PA-sugar chains*

Digestion with neuraminidase was performed in 0.1 M sodium acetate buffer, pH 5.0; with α-fucosidase in 0.1 M sodium citrate buffer, pH 6.0; and with β-galactosidase and β-N-acetylglucosaminidase in 0.01 M sodium phosphate buffer, pH 7.0, containing 1 mM $MgCl_2$. All digestions were done with 50–100 pmoles of each PA-sugar chain at 37°C for 20 h, and reactions were stopped by heating the solution at 100°C for 2 min.

*High-performance liquid chromatography (HPLC)*

The separation of PA-oligosaccharides was carried out by HPLC using a Hitachi 655A chromatograph equipped with a Rheodyne Model 7125 injector and a Hitachi Model F-1050 fluorescence spectrophotometer with reversed-phase HPLC (Cosmosil 5C18-P, 0.46 × 15 cm, Nacalai Tesque) and size-fractionation HPLC (TSK-GEL Amide-80, 0.46 × 25 cm, Tosoh Corp.) essentially according to the methods of Tomiya *et al.* (1988) and Yamamoto *et al.* (1989). In both HPLC systems, PA-oligosaccharides were detected by fluorescence using excitation and emission wavelengths of 320 and 400 nm, respectively.

**Figure 1** Sodium dodecyl sulfate polyacrylamide gel electrophoretic patterns of AFP from patients with HCC-1 **a**, HCC-2 **b**, and gallbladder carcinoma which metastasises to the liver **c**. **d**, Molecular weight marker.

**Table I** Relative yields(%) of four AFP species separated by lectin affinity chromatography with Con A and LCA

| *AFP species*[a] | *HCC-1 (patient 1)* | *HCC-2 (patient 2)* | *Gallbladder carcinoma* |
|---|---|---|---|
| Con A(+)/LCA(+) | 52 | 51 | 43 |
| Con A(+)/LCA(−) | 39 | 41 | 10 |
| Con A(−)/LCA(+) | 5 | 4 | 39 |
| Con A(−)/LCA(−) | 4 | 4 | 8 |

[a](+) and (−) represent 'reactive' and 'nonreactive', respectively.

### Results

*Preparation of four AFP species different in lectin affinity*

Purity of each patient AFP sample was established by sodium dodecyl sulfate-polyacrylamide gel electrophoresis as shown in Figure 1. The yield of AFP purification by affinity chromatography from each patient was about 90%, and the relative yields of four AFP species separated by lectin affinity chromatography with two lectin columns are listed in Table I. Purity and affinity for two lectins of each species were confirmed by crossed immunoaffinoelectrophoresis (results not shown).

Each of these four AFP species (30–100 nmoles) different in lectin affinity was subjected to hydrazinolysis to prepare PA-oligosaccharides, and subsequently PA-sugar chains from each species (50–300 pmoles) were applied to HPLC analysis. The yields of hydrazinolysis and reductive amination with 2-aminopyridine were 60–70 and 85–90%, respectively. Thus, overall yield was around 60%.

*Carbohydrate structure of Con A(+)/LCA(−) species of AFP*

The PA-oligosaccharide of the Con A(+)/LCA(−) species of AFP from HCC patient 1 was eluated at the position of the authentic PA-biantennary chain[1], both on reversed-phase HPLC (Figure 2) and on size-fractionation HPLC. by β-galactosidase and subsequent β-N-acetylglucosaminidase digestion, the elution positions were converted to that of the PA-agalactobiantennary chain, and that of the PA-trimannosyl sugar chain, respectively (results not shown). Essentially identical elution profiles of HPLC were obtained with the PA-oligosaccharide of the Con A(+)/LCA(−) species of AFP from HCC patient 2 and the patient with gallbladder carcinoma which metastasises to the liver (results not shown).

*Carbohydrate structure of Con A(+)/LCA(+) species of AFP*

The PA-oligosaccharide of the Con A(+)/LCA(+) species of AFP from HCC patient 1 was eluted at the position of the authentic PA-fucosylated biantennary chain, both on reversed-phase HPLC (Figure 3a) and on size-fractionation HPLC (Figure 4a). Upon digestion with fucosidase, the elution position was converted to that of the authentic PA-biantennary chain, as shown in Figure 3c (reversed-phase HPLC) and Figure 4c (size-fractionation HPLC). Essentially identical results were obtained for the PA-oligosaccharides of the Con A(+)/LCA(+) species of AFP from HCC patient 2 and the patient with gallbladder carcinoma which metatasises to the liver (results not shown).

Figure 2 Reversed-phase HPLC elution profile of a PA-oligosaccharide from the Con A(+)/LCA(−) species of AFP of HCC patient 1. The PA-oligosaccharide (1 in a) was eluted at the same position as that of the authentic PA-biantennary chain (1 in b).

*Carbohydrate structure of Con A(−)/LCA(+) species of AFP*

Figure 5a shows a reversed-phase HPLC elution pattern of the PA-oligosaccharide of the Con A(−)/LCA(+) species of AFP from HCC patient 1. The elution position was same as that of the authentic PA-fucosylated and N-acetylglucosaminylated biantennary chain. The elution position of Amide-80 size-fractionation HPLC was also same as that of the reference compound mentioned above (results not shown). Fucosidase digestion converted the elution position on reversed-phase HPLC of this PA-oligosaccharide to that of the PA-N-acetylglucosaminylated biantennary chain as shown in Figure 5b. Essentially identical results were obtained for the Con A(−)/LCA(+) species of AFP from HCC patient 2 and the patient with gallbladder carcinoma which metastasises to the liver (results not shown).

Figure 3 Reversed-phase HPLC elution profiles of native and fucosidase-digested PA-oligosaccharides from the Con A(+)/LCA(+) species of AFP of HCC patient 1. The native PA-oligosaccharide (1 in a) was eluted at the same position as that of the PA-fucosylated biantennary chain (1 in b). After fucosidase-digestion this PA-oligosaccharide gave a new major peak (2 in c) eluted at the position of the PA-biantennary chain (2 in d).

Figure 4 Size-fractionation HPLC elution profiles of native and fucosidase-digested PA-oligosaccharides from the Con A(+)/LCA(+) species of AFP of HCC of patient 1. The PA-oligosaccharide (1 in a) was eluted at the position of the PA-fucosylated biantennary chain (1 in b). The fucosidase-digested PA-oligosaccharide of the Con A(+)/LCA(+) species of AFP (2 in c) was eluted at the position of the PA-biantennary chain (2 in d).

**Figure 5** Reversed-phase HPLC elution profiles of native and fucosidase-digested PA-oligosaccharides from the Con A(−)/LCA(+) species of AFP of HCC patient 1. The native PA-oligosaccharide (1 in **a**) was eluted at the position of the PA-fucosylated and N-acetylglucosaminylated biantennary chain (4 in **c**). Fucosidase-digestion gave a peak (2 in **b**) at the position of the N-acetylglucosaminylated biantennary chain (peak 3 in **c**). Authentic PA-oligosaccharide standards in **c** are: 1, biantennary chain; 2, fucosylated biantennary chain; 3, N-acetylglucosaminylated biantennary chain; and 4, fucosylated and N-acetylglucosaminylated chain.

*Carbohydrate structure of Con A(−)/LCA(−) species of AFP*

Figure 6a shows an elution profile of the PA-oligosaccharides of the Con A(−)/LCA(−) species of AFP from HCC patient 1 on size-fractionation HPLC. The major peak (peak 1 in Figure 6a), representing 40 and 45% of the total peak areas in patient 1 and patient 2, respectively, was eluted at the position of the authentic PA-fucosylated triantennary chain (peak 4 in Figure 6c). The PA-triantennary oligosaccharide (peak 3 in Figure 6c) was also detected as a minor component (representing 14 and 22% of the total peak areas in the respective patient, peak 2 in Figure 5a). Upon fucosidase digestion, the major peak disappears, and an increase of the peak at the position of the authentic PA-triantennary chain, was observed (peak 2 in Figure 6b).

To confirm the chemical structure of the sugar chain, the main peak oligosaccharide was collected, and a portion was subjected to fucosidase digstion. Figure 7a shows an elution profile of the PA-oligosaccharide thus isolated on size-fractionation HPLC. The elution position (peak 1 in Figure 7a) was same as that of the PA-fucosylated triantennary chain (peak 6 in Figure 7c). Fucosidase digestion gave an oligosaccharide eluted at a position (peak 2 in Figure 7b) of the authentic PA-triantennary chain (peak 5 in Figure 7c). the fucosidase-treated oligosaccharide was collected and then subjected to β-galactosidase digestion and subsequently to β-N-acetylglucosaminidase digestion. The elution positions of resultant oligosaccharides (peak 3 in Figure 7d and peak 4 in Figure 7e) were identical with those of authentic oligosaccharides, a PA-agalactotriantennary chain (peak 2 in Figure 7f), and a PA-trimannosyl chain (peak 1 in Figure 7f) standards, respectively.

The elution profile of the PA-oligosaccharide of the Con A(−)/LCA(−) species of AFP from HCC patient 1 by reversed-phase HPLC is shown in Figure 8. By this reversed-phase HPLC with Cosmosil 5C18-P, the PA-oligosaccharide of the N-acetylglucosaminylated biantennary chain and that of the fucosylated triantennary chain were eluted at almost the same position (peak 4 in Figure 8c and peak 5 in Figure 8d), and therefore it was difficult to know their presence or absence. However, when digested with fucosidase, a major peak (2 in Figure 8b) was shifted to the position of the PA-triantennary chain (peak 3 in Figure 8c). A minor peak (1 in Figure 8b) remaining at the initial position after repeated fucosidase digestions was considered to represent N-acetylglucosaminylated biantennary chain (peak 5 in Figure 8d). These results indicated that major components of PA-oligosaccharides of Con A(−)/LCA(−) species of AFP from the patient with HCC was the fucosylated triantennary chain. Although it was difficult to identify each peak, several minor components eluted at faster positions than that of the major

**Figure 6** Size-fractionation HPLC elution profiles of native and fucosidase-digested PA-oligosaccharides of the Con A(−)/LCA(−) species of AFP of patient 1. The major peak (1 in **a**, 40% of the total peak area) was eluted at the position of the fucosylated triantennary chain (4 in **c**). The PA-triantennary chain was also detected as a minor component (2 in **a**, 14% of the total peak area). By fucosidase digestion, the main peak at the elution position of the PA-fucosylated triantennary chain disappeared, and an increase of a peak (2 in **b**) at the position of PA-triantennary chain was observed. Authentic PA-oligosaccharide standards in **c** are: 1, biantennary chain; 2, fucosylated biantennary chain; 3, triantennary chain; and 4, fucosylated triantennary chain.

**Figure 7** Size-fractionation HPLC elution profiles of the isolated PA-oligosaccharide (peak 1 in Figure 5a) and its stepwise exoglycosidase digest. The elution position of the native one (1 in **a**) was same as that of the fucosylated triantennary chain (6 in **c**), and α-fucosidase digestion gave a peak (2 in **b**) corresponding to the triantennary chain (5 in **b**). The oligosaccharide of this peak was collected and subjected to β-galactosidase digestion and subsequently to β-N-actylglucosaminidase digestion. The β-galactosidase digest gave a peak (3 in **d**) corresponding to the agalactotriantennary chain (2 in **f**), and β-N-acetyl-glucosaminidase digest gave a peak (4 in **e**) at the position of the trimannosyl chain (1 in **f**). Authentic PA-oligosaccharide standards in **c** and **f** are: 1, trimannosyl chain; 2, agalactotriantennary chain; 3, biantennary chain; 4, fucosylated biantennary chain; 5, triantennary chain; and 6, fucosylated triantennary chain.

component (Figure 6a), apepared to be fucosylated species, because after fucosidase digestion these minor peaks disappeared and new peaks became detectable at faster position than that of untreated minor components (Figure 6b).

Essentially the same elution profiles on HPLC were obtained for Con A(−)/LCA(−) species of AFP from HCC patient 2 and the patient with gallbladder carcinoma which metastasises to the liver. However, the ratio (10.1) of the fucosylated to non-fucosylated triantennary sugar chains of AFP from the patient with gallbladder carcinoma which metastasises to the liver was much higher than those (3.2 for patient 1 and 1.9 for patient 2) in patients with HCC. The peak areas due to the fucosylated and the nonfucosylated triantennary chains in this disease represented 32 and 3% of the total area, respectively.

### Discussion

It is now accepted that there are several molecular species of AFP with different affinity for Con A and LCA. LCA binds specifically the biantennary chain with a fucose residue at the innermost N-acetylglucosamine residue at the trimannosyl core (fucosylated biantennary chain) and that both with a fucose and a bistecting-N-acetylglucosamine residue (fucosylated and N-acetylglucosaminylated biantennary chain) (Kornfeld *et al.*, 1981). However, if these biantennary structures undergo modification of further branching leading to triantennary structures, they become nonreactive with LCA (Montreuil *et al.*, 1983). On the other hand, Con A binds specifically the biantennary and the fucosylated biantennary chains. This binding does not occur if the biantennary sugar chain structure undergoes modification of bisecting-glucosaminylation and/or further branching leading to the formation of the triantennary and tetraantennary structures (Baenziger & Fiete, 1979).

On the basis of these facts, we have proposed the carbohydrate structures of various AFP species with difference in affinity for Con A and LCA (Aoyagi *et al.*, 1985, 1991; Kornfeld *et al.*, 1981; Montreuil *et al.*, 1983; Baenziger *et al.*, 1979), but they remain to be confirmed.

Recently, a very sensitive and convenient method has been developed to study the fine carbohydrate structures by combination of derivatisation into fluorescent oligosaccharide and separation by HPLC (Hase *et al.*, 1984; Tomiya *et al.*, 1988; Yamamoto *et al.*, 1989; Nishiura *et al.*, 1990). Since a number of PA-oligosaccharide, the structures of which have been established by $^{1}$H-nuclear magnetic resonance, have become commercially available, we applied this method to study the fine structures of various AFP species.

The present results are summarised in Table II. Here, we propose the classification of carbohydrate structures of human AFP according to the reactivity with lectins and the disease category (Table II). The first is the Con A(+)/LCA(−) sugar chain, and the structure is of the biantennary complex type, a basic carbohydrate structure of AFP. This structure is predominantly observed in AFP of nonneoplastic liver diseases. The second is the Con A(+)/LCA(+) chain composed of the fucosylated biantennary complex type, which is predominantly observed in AFP of HCC. The third is the Con A(−)/LCA(+) chain which is the biantennary complex type with fucosylation and bisecting-N-acetylglucosaminylation, and this type is observed predominantly in AFP of carcinomas of digestive organs which metastasise to the liver and yolk sac tumour. The fourth is the Con A(−)/LCA(−) chain which includes three kinds of carbohydrate structure, namely, the biantennary complex type with bisecting-acetylglucosaminylation, the triantennary complex type and the triantennary complex type with fucosylation. The

**Figure 8** Reversed-phase HPLC elution profiles of native **a**, and fucosidase-digested PA-oligosaccharides **b**, from the Con A(−)/LCA(−) species of AFP of HCC patient 1. The native oligosaccharides gave a major peak (1 in **a**) at the position of the authentic fucosylated triantennary chain (4 in **c**). In this system, the N-acetylglucosaminylated biantennary chain (5 in **d**) was also eluted at the same position. Fucosidase digestion resulted in an increase of a peak (2 in **b**) at the position of triantennary chain (3 in **c**). A residual minor peak at the initial position (peak 1 in **b**) was considered to the N-acetylglucosaminylated biantennary chain (5 in **d**). Authentic PA-oligosaccharides in **c** and **d** are: 1, biantennary chain; 2, fucosylated biantennary chain; 3, triantennary chain; 4, fucosylated triantennary chain; 5, N-acetylglucosaminylated biantennary chain; and 6, fucosylated and N-acetylglucosaminylated biantennary chain.

N-acetyl-glucosaminylated biantennary chain is predominantly observed in AFP of carcinoma which metastasises to the liver and yolk sac tumour. The triantennary chain and its fucosylated form are present in AFP of HCC, carcinoma which metastasises to the liver and yolk sac tumour.

Recently we reported that the measurement of a fucosylation index of AFP is useful for distinction between HCC and nonneoplastic diseases of the liver (Aoyagi *et al.*, 1984, 1991). The fucosylation index of AFP was defined as the percentage of the LCA-reactive species in total AFP (Aoyagi *et al.*, 1991). The present study revealed that more than the 40% of Con A-nonreactive species of AFP were fucosylated, even if it is not reactive with LCA. In our previous study the percentage of the Con A-nonreactive species of AFP was 5 ± 7% in 351 patients with HCC (Aoyagi *et al.*, 1991) and we may expect a little effect of this species on the fucosylation index. Special consideration, however, should be given to evaluate the fucosylation index determined by crossed immunoaffinoelectrophoresis with Con A and LCA, when the percentage of the Con A nonreactive species is high.

Thus, evidence presented here and our previous study indicate that increases in fucosylation and in branching to form new antennae are observed in carbohydrate chains of AFP from patients with neoplastic diseases of the liver. Other studies also indicated the tumour associated increase in fucosylation and branching. For example, Campion *et al.* (1989) reported the presence of fucosylated triantennary, tetraantennary and pentaantennary glycans, in transferrin synthesised by the human hepatocarcinoma cell line Hep G2. Dennis *et al.* (1987, 1989) reported that transfection of an activated *ras* gene into the non-metastatic line of SP1 mouse mammary tumour cells resulted in the induction of both metastatic potential and elevated levels of β 1-6 branched oligosaccharides and that oncogenes conferring metastatic potential induced increased branching of asparagine-linked oligosaccharides in rat2 fibroblasts. However, the biological significance of this tumour-associated change remains to be clarified.

Some of chemical structures of sugar chains of human AFP have already been reported by Yoshima *et al.* (1980) and Yamashita *et al.* (1983). However, carbohydrate structures in the molecular species of human AFP with different affinity for Con A and LCA were not fully investigated. These authors presented the chemical structures of sugar chains of human AFP from ascites fluids as biantennary, fucosylated biantennary, N-acetylglucosaminylated biantennary and fucosylated, N-acetylglucoaminylated chains. These structures were confirmed to be present in our AFP preparations.

On the other hand, they reported that human AFP did not contain the triantennary chain. However, our present study clearly indicated the presence of the triantennary chains with and without a fucose residue at the innermost N-acetylglucosamine residue in AFP from patients with neoplastic diseases of the liver. Thus, this is the first report to establish the presence of these structures in detail in human AFP.

**Table II** Classification of carbohydrate structures of AFP and their reactivity to lectins with special reference to disease category

| *Lectin reactivity*[a] | *Carbohydrate structure* | *Disease category*[b] |
|---|---|---|
| Con A(+)/LCA(−) | Biantennary sugar chain | Non-neoplastic liver diseases |
| Con A(+)/LCA(+) | Fucosylated biantennary sugar chain | Hepatocellular carcinoma |
| Con A(−)/LCA(+) | Fucosylated and N-acetyl-glucosaminylated biantennary sugar chain | Carcinomas of digestive organs which metastasise to the liver and yolk sac tumour |
| Con A(−)/LCA(−) | N-Acetylglucosaminylated biantennary sugar chain | Carcinomas of digestive organs which metastasise to the liver and yolk sac tumour |
| | Triantennary and fucosylated triantennary sugar chains | Hepatocellular carcinoma, carcinomas of digestive organs which metastasise to the liver and yolk sac tumour |

[a](+) and (−) represent 'reactive' and 'nonreactive', respectively. [b]Disease in which AFP with the relevant carbohydrate structure is found characteristically.

In the case of AFP of foetal calf serum, Krusius and Ruoslahti (1982) found the same structure at the triantennary chain reported here. Bayard et al. (1983) showed that the presence in rat AFP of a similar species to our N-acetylglucosaminylated biantennary chain. Although the precise molecular basis for these variations is not clear at present, it is conceivable to assume that the difference in a set of sugar transferases expressed would lead to the qualitative and quantitative differences in each molecular species of AFP.

This study was supported in part by a Grant-in-Aid from The Niigata University Science Foundation.

### References

ABELEV, G.I. (1968). Production of embryonal serum alpha-globulin by hepatomas: review of experimental and clinical data. *Cancer Res.*, **28**, 1344–1355.

ALPERT, E. & FELLER, E.R. (1978). Alpha-fetoprotein (AFP) in benign liver diseases. Evidence that normal liver regeneration does not induce AFP synthesis. *Gastroenterology*, **74**, 856–858.

ALPERT, E., PINN, V.W. & ISSELBACHER, K.J. (1971). Alpha-fetoprotein in a patient with gastric carcinoma metastatic to the liver. *N. Engl. J. Med.*, **285**, 1058–1059.

AOYAGI, Y., IKENAKA, T. & ICHIDA, F. (1977). Comparative chemical structures of human α-fetoproteins from fetal serum and from fluid of a patient with hepatoma. *Cancer Res.*, **37**, 3663–3667.

AOYAGI, Y., ISEMURA, M., YOSIZAWA, Z., SUZUKI, Y., SEKINE, C., ONO, T. & ICHIDA, F. (1985). Fucosylation of serum alpha-fetoprotein with primary hepatocellular carcinoma. *Biochim. Biophys. Acta.*, **830**, 217–223.

AOYAGI, Y., SUZUKI, Y., IGARASHI, K., SAITOH, A., OGURO, M., YOKOTA, T., MORI, S., NOMOTO, M., ISEMURA, M. & ASAKURA, H. (1991). The usefulness of the simultaneous determination of glucosaminylation and fucosylation indices of alpha-fetoprotein in the differential diagnosis of neoplastic diseases of the liver. *Cancer*, **67**, 2390–2394.

AOYAGI, Y., SUZUKI, Y., ISEMURA, M., SOGA, K., OZAKI, T., ICHIDA, T., INOUE, K., SASAKI, H. & ICHIDA, F. (1984). Differential reactivity of alpha-fetoprotein with lectins and evaluation of its usefulness in the diagnosis of hepatocellular carcinoma. *Gann*, **75**, 809–815.

BAENZIGER, J.U. & FIETE, D. (1979). Structural determinations of concanavalin A specificity for oligosaccharides. *J. Biol. Chem.*, **254**, 2400–2407.

BAYARD, B., KERCKAERT, J.P., STRECKER, G., DOPLAND, L., VAN HALBEEK, H. & VLIEGENTHART, J.F.G. (1983). Structure determination of the carbohydrate chains of rat α-feotprotein. *Eur. J. Biochem.*, **137**, 319–323.

BREBOROWICZ, J., MACKIEWICZ, A. & BREBOROWICZ, D. (1981). Microheterogeneity of alpha-fetoprotein in patient serum as demonstrated by lectin affino-electrophoresis. *Scand. J. Immunol.*, **14**, 15–20.

CAMPION, B., LEGER, D., WIERUSZESKI, J.M., MONTREUIL, J. & SPIK, G. (1989). Presence of fucosylated triantennary, tetraantennary and pentaantennary glycans in transferrin synthesized by the human hepatocarcinoma cell line Hep G2. *Eur. J. Biochem.*, **184**, 405–413.

DENNIS, J.W., KOSH, K., BRYCE, D.M. & BRITEMAN, M.L. (1989). Oncogenes conferring metastatic potential induced increased branching of Asn-linked oligosaccharides in rat2 fibroblasts. *Oncogene*, **4**, 853–860.

DENNIS, J.L., LAFERTE, S., WAGHORNE, C., BREITMAN, M.L. & KERBEL, R.S. (1987). β1-6 Branching of Asn-linked oligosaccharides is directly associated with metastasis. *Science*, **236**, 582–585.

HASE, S., IBUKI, T. & IKENAKA, T. (1984). Reexamination of the pyridylamination used for fluorescence labeling of oligosaccharides and its application to glycoproteins. *J. Biochem.*, **95**, 197–203.

ISHIGURO, T., SUGITACHI, I., SAKAGUCHI, H. & ITANI, S. (1985). Serum alpha-fetoprotein subfraction in patients with primary hepatoma or hepatic metastasis of gastric cancer. *Cancer*, **55**, 156–159.

KARVOUNTZIS, G.G. & REDECKER, A.G. (1974). Relation of alpha-fetoprotein in acute hepatitis to severity and prognosis. *Intern. Med.*, **80**, 156–160.

KORNFELD, K., REITMAN, M.L. & KORNFELD, R. (1981). The carbohydrate-binding specificity of pea and lentil lectins. *J. Biol. Chem.*, **256**, 6633–6640.

KRUSIUS, T. & RUOSLAHTI, E. (1982). Carbohydrate structure of the concanavalin A molecular variants of alpha-fetoprotein. *J. Biol. Chem.*, **257**, 3453–3457.

LAEMMLI, U.K. (1970). Cleavage of the structural proteins during assembly of the head of bacteriophage T4. *Nature*, **227**, 680–685.

MCINTIRE, K.R., WALDMANN, T.A., MOERTEL, C.G. & GO, V.L.W. (1975). Serum alpha-fetoprotein in patients with neoplasms of the gastrointestinal tract. *Cancer Res.*, **35**, 991–996.

MONTREUIL, J., DEBRAY, P., DEBEIRE, P. & DELANNOY, P. (1983). Lectins as oligosaccharide receptors. In *Structural Carbohydrates in the Liver*. Popper, H., Reutter, W., Köttgen, E. & Gudat, F. (eds). pp. 239–258. MTP Press Limited: Boston, The Hauge, Dordrecht, Lancaster.

NISHI, S. & HIRAI, H. (1973). Radioimmunoassay of alpha-fetoprotein in hepatoma, other liver diseases, and pregnancy. *Gann Monogr.*, **14**, 79–87.

NISHIURA, T., FUJII, S., KANAYAMA, Y., NISHIKAWA, A., TOMIYAMA, Y., IIDA, M., KARASUNO, T., NAKAO, H., YONEZAWA, T., TANIGUCHI, N. & TARUI, S. (1990). Carbohydrate analysis of immunoglobulin G myeloma proteins by lectin and high performance liquid chromatography: role of glycosyltransferases in the structures. *Cancer Res.*, **50**, 5345–5350.

O'CONOR, G.I., TATARINOV, Y.S., ABELEV, G.I. & URIEL, J. (1970). A collaborative study for the evaluation of a serologic test for primary liver cancer. *Cancer*, **25**, 1091–1098.

RUOSLAHTI, E., ENGVALL, E., PEKKALA, A. & SEPPÄLÄ, M. (1978). Developmental changes in carbohydrate moiety of human alpha-fetoprotein. *Int. J. Cancer*, **22**, 515–520.

TAKETA, K. & HIRAI, H. (1989). Lectin affinity electrophoresis of alpha-fetoprotein in cancer diagnosis. *Electrophoresis*, **10**, 562–567.

TOMIYA, N., AWAYA, J., KURONO, M., ENDO, S., ARATA, T. & TAKAHASHI, N. (1988). Analysis of N-linked oligosaccharides using a two-dimensional mapping technique. *Anal. Biochem.*, **171**, 73–90.

YAMAMOTO, S., HASE, S., FUKUDA, S., SANO, O. & IKENAKA, T. (1989). Structures of the sugar chain of interferon-γ produced by human myelomonocyte cell line HBL-38. *J. Biochem.*, **105**, 547–555.

YAMASHITA, K., HITOI, A., TSUCHIDA, Y., NISHI, S. & KOBATA, A. (1983). Sugar chain of alpha-fetoprotein produced in human yolk sac tumor. *Cancer Res.*, **43**, 4691–4695.

YOSHIMA, H., MIZUOCHI, T., ISHII, M. & KOBATA, A. (1980). Structure of the asparagine-linked sugar chains of alpha-fetoprotein purified from human ascites fluid. *Cancer Res.*, **40**, 4276–4281.


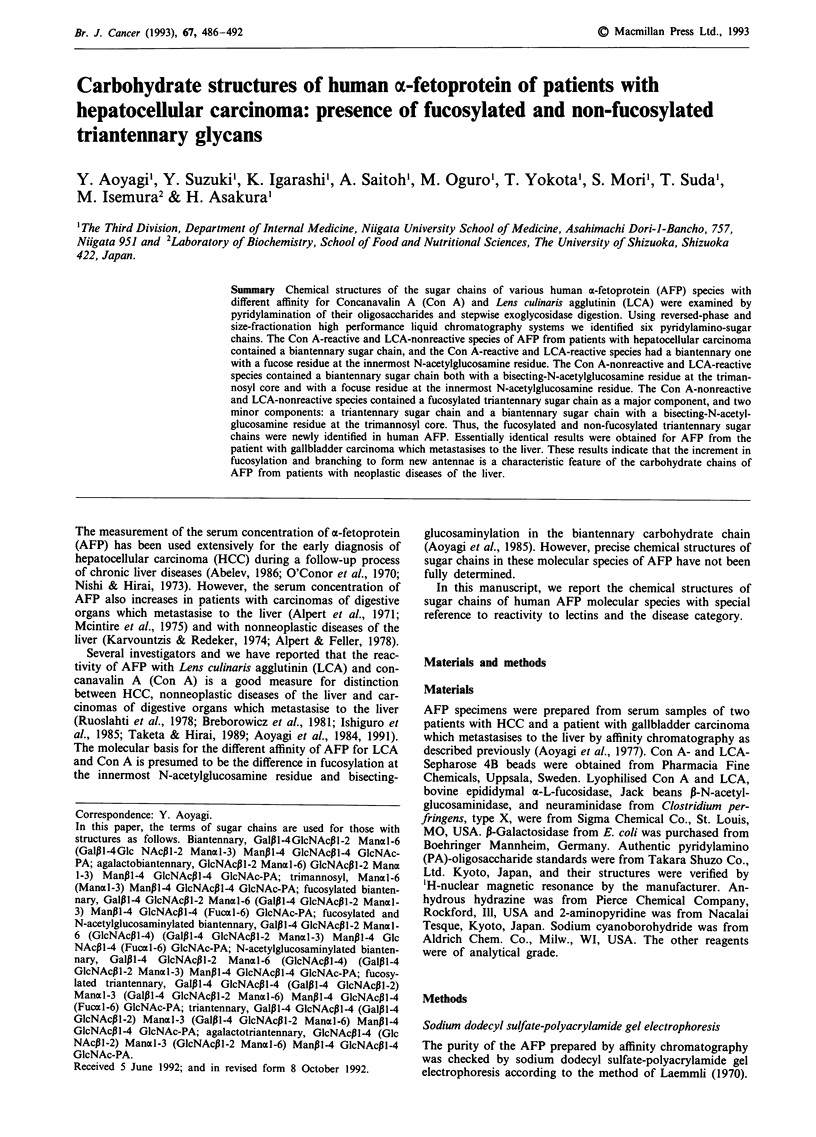

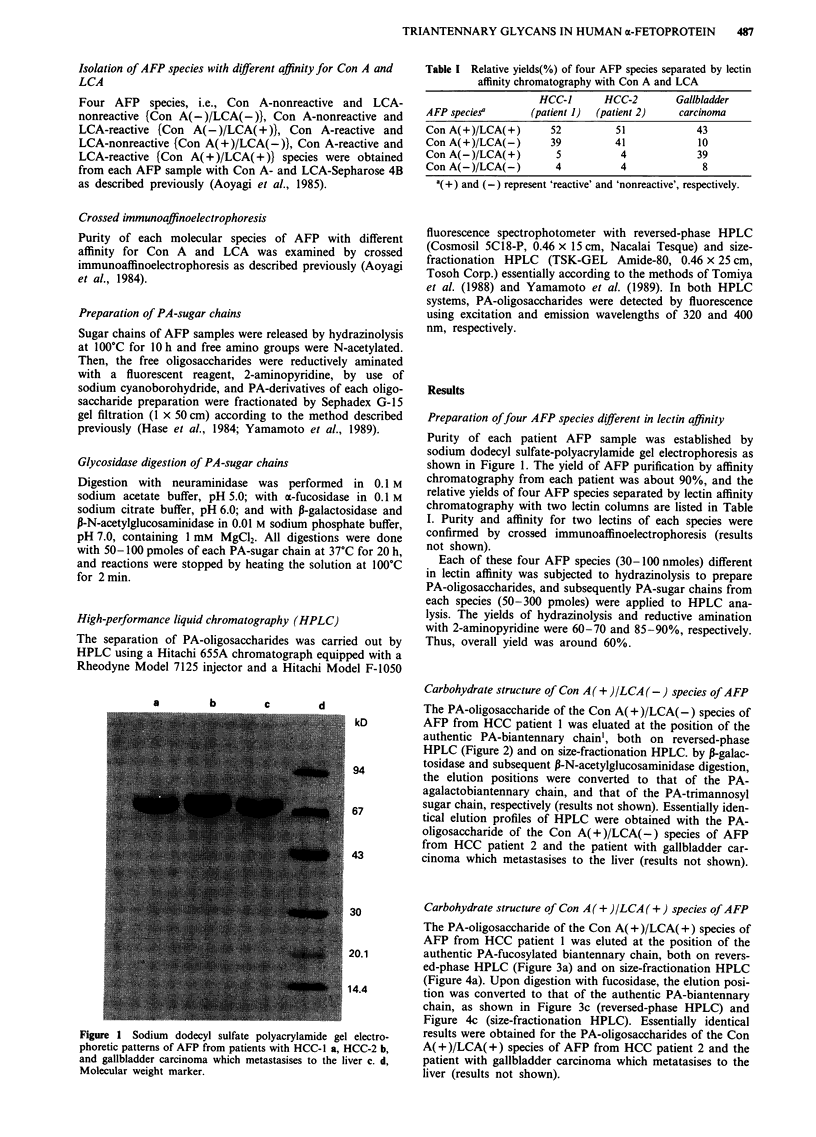

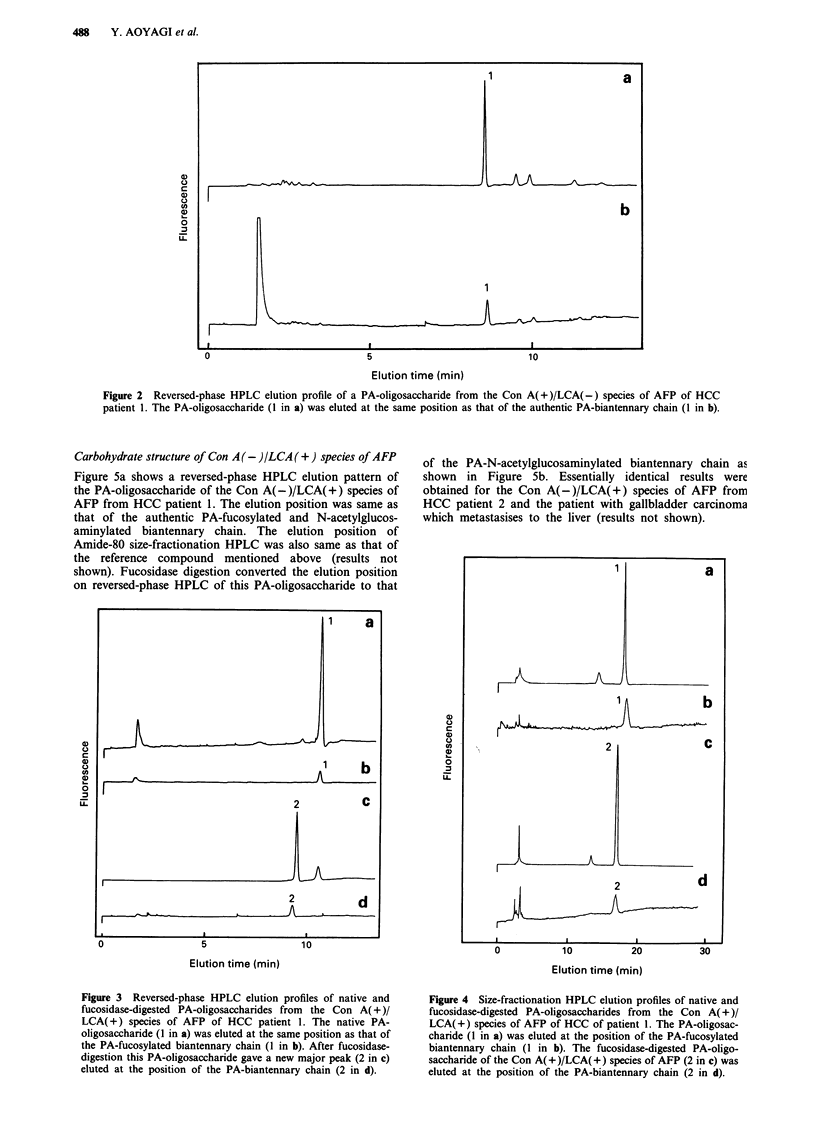

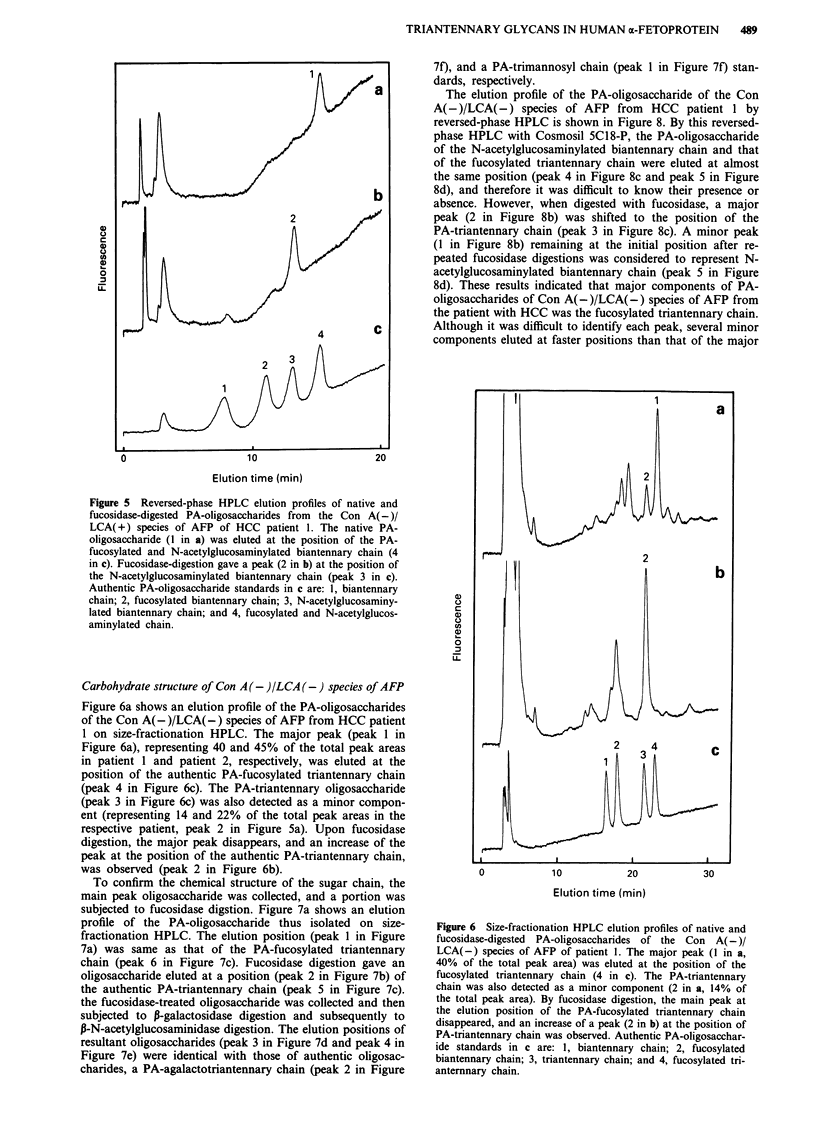

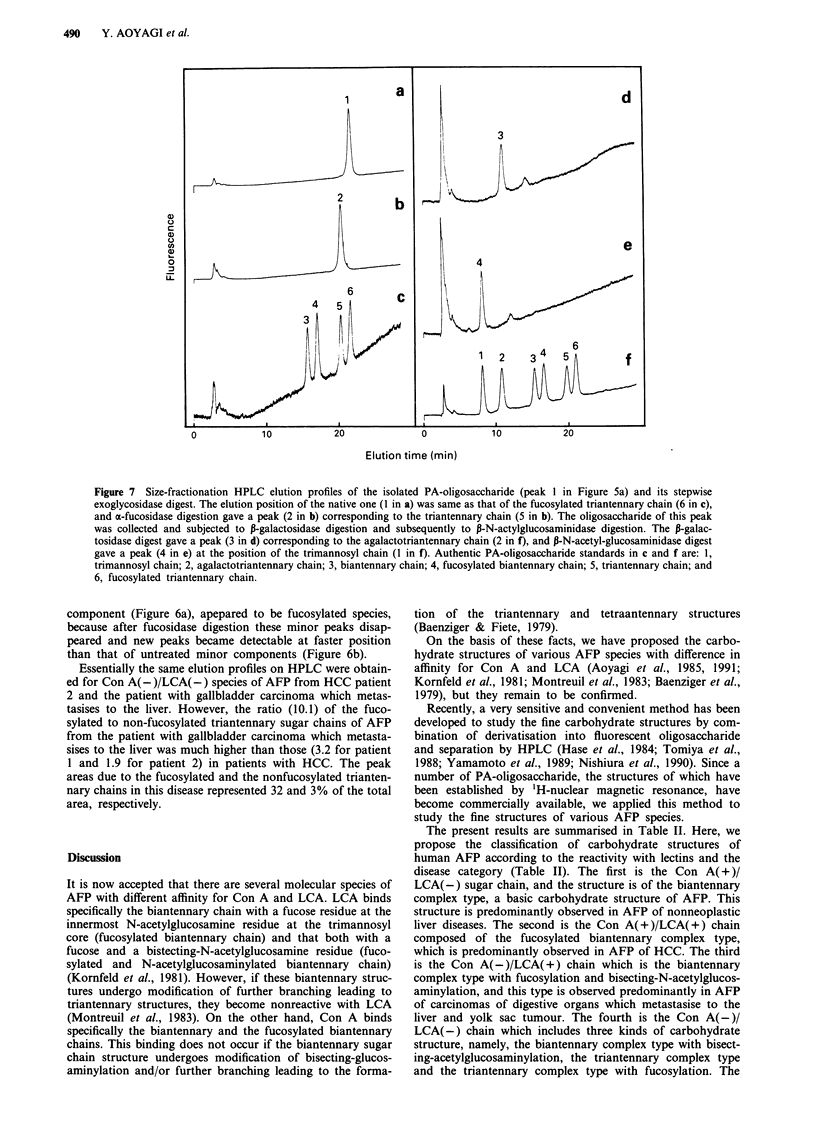

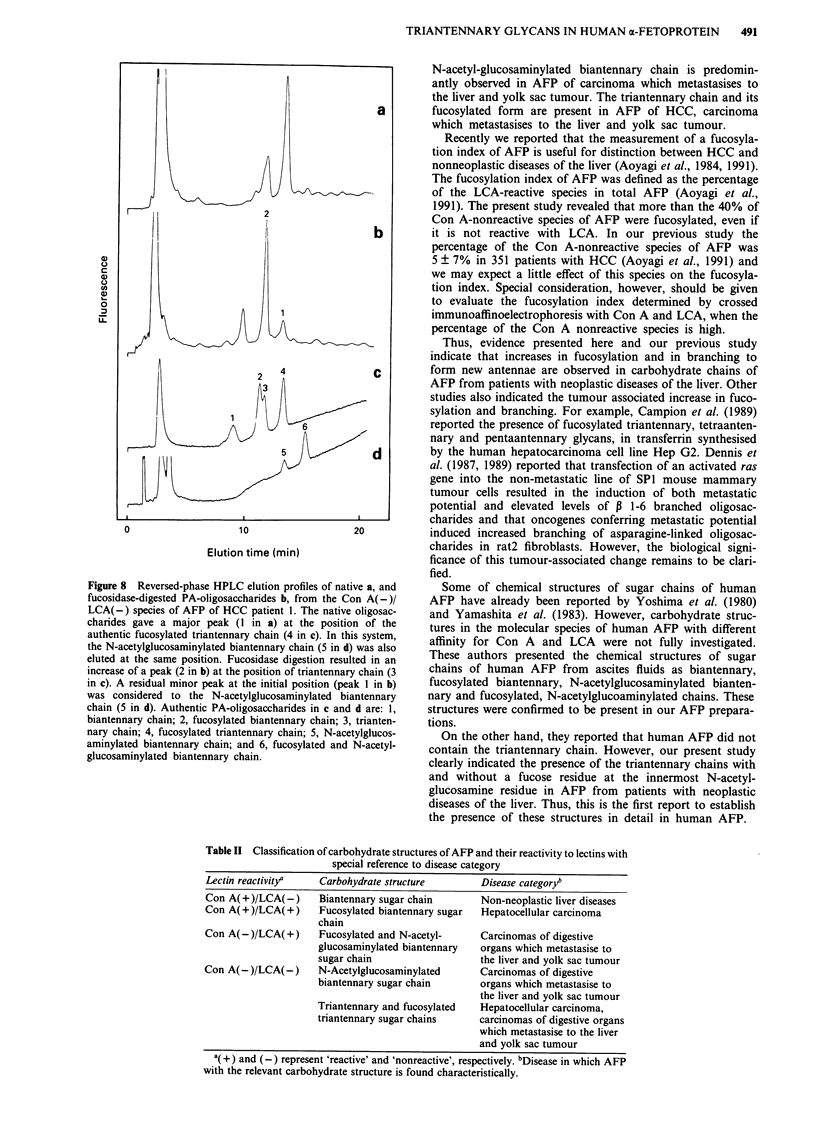

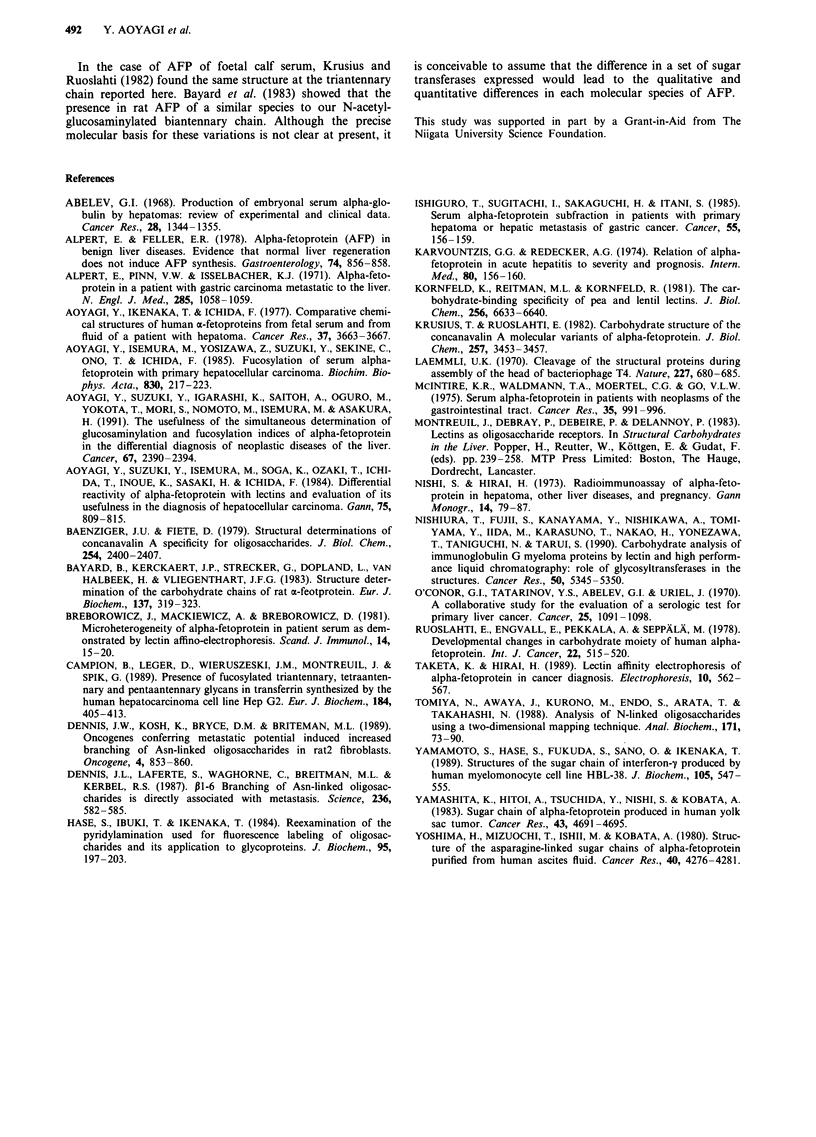


## References

[OCR_00756] Abelev G. I. (1968). Production of embryonal serum alpha-globulin by hepatomas: review of experimental and clinical data.. Cancer Res.

[OCR_00761] Alpert E., Feller E. R. (1978). Alpha-fetoprotein (AFP) in benign liver disease. Evidence that normal liver regeneration does not induce AFP synthesis.. Gastroenterology.

[OCR_00765] Alpert E., Pinn V. W., Isselbacher K. J. (1971). Alpha-fetoprotein in a patient with gastric carcinoma metastatic to the liver.. N Engl J Med.

[OCR_00770] Aoyagi Y., Ikenaka T., Ichida F. (1977). Comparative chemical structures of human alpha-fetoproteins from fetal serum and from ascites fluid of a patient with hepatoma.. Cancer Res.

[OCR_00774] Aoyagi Y., Isemura M., Yosizawa Z., Suzuki Y., Sekine C., Ono T., Ichida F. (1985). Fucosylation of serum alpha-fetoprotein in patients with primary hepatocellular carcinoma.. Biochim Biophys Acta.

[OCR_00780] Aoyagi Y., Suzuki Y., Igarashi K., Saitoh A., Oguro M., Yokota T., Mori S., Nomoto M., Isemura M., Asakura H. (1991). The usefulness of simultaneous determinations of glucosaminylation and fucosylation indices of alpha-fetoprotein in the differential diagnosis of neoplastic diseases of the liver.. Cancer.

[OCR_00790] Aoyagi Y., Suzuki Y., Isemura M., Soga K., Ozaki T., Ichida T., Inoue K., Sasaki H., Ichida F. (1984). Differential reactivity of alpha-fetoprotein with lectins and evaluation of its usefulness in the diagnosis of hepatocellular carcinoma.. Gan.

[OCR_00795] Baenziger J. U., Fiete D. (1979). Structural determinants of concanavalin A specificity for oligosaccharides.. J Biol Chem.

[OCR_00800] Bayard B., Kerckaert J. P., Strecker G., Dorland L., van Halbeek H., Vliegenthart J. F. (1983). Structure determination of the carbohydrate chains of rat alpha-fetoprotein.. Eur J Biochem.

[OCR_00806] Breborowicz J., Mackiewicz A., Breborowicz D. (1981). Microheterogeneity of alpha-fetoprotein in patient serum as demonstrated by lectin affino-electrophoresis.. Scand J Immunol.

[OCR_00812] Campion B., Léger D., Wieruszeski J. M., Montreuil J., Spik G. (1989). Presence of fucosylated triantennary, tetraantennary and pentaantennary glycans in transferrin synthesized by the human hepatocarcinoma cell line Hep G2.. Eur J Biochem.

[OCR_00819] Dennis J. W., Kosh K., Bryce D. M., Breitman M. L. (1989). Oncogenes conferring metastatic potential induce increased branching of Asn-linked oligosaccharides in rat2 fibroblasts.. Oncogene.

[OCR_00825] Dennis J. W., Laferté S., Waghorne C., Breitman M. L., Kerbel R. S. (1987). Beta 1-6 branching of Asn-linked oligosaccharides is directly associated with metastasis.. Science.

[OCR_00831] Hase S., Ibuki T., Ikenaka T. (1984). Reexamination of the pyridylamination used for fluorescence labeling of oligosaccharides and its application to glycoproteins.. J Biochem.

[OCR_00837] Ishiguro T., Sugitachi I., Sakaguchi H., Itani S. (1985). Serum alpha-fetoprotein subfractions in patients with primary hepatoma or hepatic metastasis of gastric cancer.. Cancer.

[OCR_00843] Karvountzis G. G., Redeker A. G. (1974). Relation of alpha-fetoprotein in acute hepatitis to severity and prognosis.. Ann Intern Med.

[OCR_00848] Kornfeld K., Reitman M. L., Kornfeld R. (1981). The carbohydrate-binding specificity of pea and lentil lectins. Fucose is an important determinant.. J Biol Chem.

[OCR_00853] Krusius T., Ruoslahti E. (1982). Carbohydrate structure of the concanavalin A molecular variants of alpha-fetoprotein.. J Biol Chem.

[OCR_00858] Laemmli U. K. (1970). Cleavage of structural proteins during the assembly of the head of bacteriophage T4.. Nature.

[OCR_00861] McIntire K. R., Waldmann T. A., Moertel C. G., Go V. L. (1975). Serum alpha-fetoprotein in patients with neoplasms of the gastrointestinal tract.. Cancer Res.

[OCR_00880] Nishiura T., Fujii S., Kanayama Y., Nishikawa A., Tomiyama Y., Iida M., Karasuno T., Nakao H., Yonezawa T., Taniguchi N. (1990). Carbohydrate analysis of immunoglobulin G myeloma proteins by lectin and high performance liquid chromatography: role of glycosyltransferases in the structures.. Cancer Res.

[OCR_00886] O'Conor G. T., Tatarinov Y. S., Abelev G. I., Uriel J. (1970). A collaborative study for the evaluation of a serologic test for primary liver cancer.. Cancer.

[OCR_00891] Ruoslahti E., Engvall E., Pekkala A., Seppälä M. (1978). Developmental changes in carbohydrate moiety of human alpha-fetoprotein.. Int J Cancer.

[OCR_00896] Taketa K., Hirai H. (1989). Lectin affinity electrophoresis of alpha-fetoprotein in cancer diagnosis.. Electrophoresis.

[OCR_00901] Tomiya N., Awaya J., Kurono M., Endo S., Arata Y., Takahashi N. (1988). Analyses of N-linked oligosaccharides using a two-dimensional mapping technique.. Anal Biochem.

[OCR_00907] Yamamoto S., Hase S., Fukuda S., Sano O., Ikenaka T. (1989). Structures of the sugar chains of interferon-gamma produced by human myelomonocyte cell line HBL-38.. J Biochem.

[OCR_00913] Yamashita K., Hitoi A., Tsuchida Y., Nishi S., Kobata A. (1983). Sugar chain of alpha-fetoprotein produced in human yolk sac tumor.. Cancer Res.

[OCR_00918] Yoshima H., Mizuochi T., Ishii M., Kobata A. (1980). Structure of the asparagine-linked sugar chains of alpha-fetoprotein purified from human ascites fluid.. Cancer Res.

